# Complete remission of advanced pancreatic cancer induced by claudin18.2-targeted CAR-T cell therapy: a case report

**DOI:** 10.3389/fimmu.2024.1325860

**Published:** 2024-02-29

**Authors:** Guocheng Zhong, Xiaomin Zhang, Zheng Guo, Yujie Gao, Bochen Zhao, Xianhao Liu, Lei Chen, Jingqiao Qiao, Chuan Yu, Lixin Wang, Yisheng Li, Li Yu

**Affiliations:** ^1^ Department of Hematology and Oncology, Shenzhen University General Hospital, International Cancer Center, Shenzhen Key Laboratory, Hematology Institution of Shenzhen University, Shenzhen University Health Science Center, Shenzhen University, Shenzhen, China; ^2^ Guangdong Key Laboratory for Biomedical Measurements and Ultrasound Imaging, National-Regional Key Technology Engineering Laboratory for Medical Ultrasound, School of Biomedical Engineering, Shenzhen University Medical School, Shenzhen, China; ^3^ R&D Department, Shenzhen Haoshi Biotechnology Co., Ltd, Shenzhen, China; ^4^ Biomedical Laboratory, Shenzhen University-Haoshi Cell Therapy Institute, Shenzhen, China

**Keywords:** CAR-T cell therapy, advanced pancreatic cancer, claudin18.2, complete remission, antigen-negative relapse

## Abstract

Pancreatic cancer (PC) is one of the most malignant tumors in digestive system due to its highly invasive and metastatic properties. At present, conventional treatment strategies for PC show the limited clinical efficacy. Therefore, novel effective therapeutic strategies are urgently needed. Here, we report a case of complete remission of advanced PC induced by claudin18.2-targeted CAR-T cell therapy. The patient was a 72-year-old man who was diagnosed with pancreatic ductal adenocarcinoma 2 years ago, and he experienced tumor recurrence and multiple metastases after pancreaticoduodenectomy and multi-line chemotherapies, including liver, peritoneum, and cervical lymph node metastases. Then, the patient was referred to our department for further treatment of metastatic PC, and he was enrolled in a clinical trial of claudin18.2-targeted CAR-T cell therapy. After lymphodepleting chemotherapy, the patient received claudin18.2-targeted CAR-T cell infusion at a dose of 1.2 × 10^6^ cells/kg on November 21, 2022. During CAR-T cell therapy, the patient experienced grade 2 cytokine release syndrome (CRS) and gastric mucosa injury, which were controlled by tocilizumab and conventional symptomatic and supportive treatment. The patient achieved a complete response (CR) 1 month after claudin18.2-targeted CAR-T cell therapy, and remained in clinical remission for 8 months. Unfortunately, the patient experienced claudin18.2-negative relapse in July, 2023. Despite antigen-negative relapse after claudin18.2-targeted CAR-T cell infusion, the patient achieved sustained remission for 8 months, which indicates that claudin18.2-targeted CAR-T cell therapy is an extremely effective therapeutic strategy for the treatment of advanced PC.

## 1 Introduction

Pancreatic cancer (PC), commonly known as the “king of cancers”, is one of the most lethal malignant tumors with a 5-year survival rate of less than 10% (1). Due to the lack of effective screening, most of the patients with PC usually present with advanced or metastatic disease at diagnosis. The efficacy of conventional treatment for PC, including chemotherapy, radiotherapy, and immune checkpoint inhibitors, is frequently unsatisfactory. Moreover, PC is prone to recurrence and metastasis even in patients who have undergone surgery, so the prognosis of PC is extremely poor (2, 3). Therefore, the novel effective therapeutic strategies for PC are desperately needed.

Chimeric antigen receptor T (CAR-T) cell therapy is a highly promising adoptive T cell therapy, especially in cancer immunotherapy. In recent years, it has achieved remarkable success in hematological malignancies, including relapsed/refractory non-Hodgkin lymphoma, B-cell acute lymphocytic leukemia, and multiple myeloma, with a cure rate of more than 80% in multiple clinical trials. Considering the astounding efficacy of CAR-T cell therapy in hematological malignancies, CAR-T cell therapy is also being explored in solid tumors at present. Claudin18.2, an isoform of claudin18, is a member of tight junction proteins, and its expression in normal tissues is strictly limited to differentiated epithelial cells of the gastric mucosa. However, during carcinogenesis, loss of cell polarity results in the exposure of claudin18.2 epitopes, such as gastric cancer, gastroesophageal junction cancer, and PC. Thus, claudin18.2 is highly expressed in these malignant tumors (4, 5). In addition, claudin 18.2 is also expressed in distant tumor metastasis, and involved in the proliferation, differentiation and migration of tumor cells (5). More importantly, the positive rate of claudin18.2 antigen in PC cases is over 50%, and the vast majority of positive cases were characterized by high expression of claudin18.2 (6, 7). Therefore, claudin 18.2 is an ideal target for CAR-T cell therapy in metastatic or advanced PC (8–10). Herein, we present a case of complete remission of advanced PC induced by claudin18.2-targeted CAR-T cell therapy.

## 2 Case presentation

**Figure 1:**
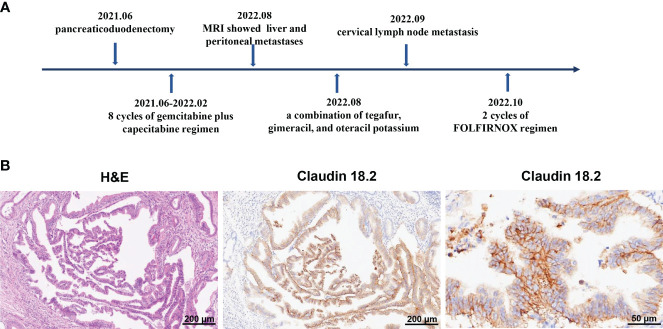
**(A)** Timeline of multi-line therapies before claudin18.2-targeted CAR-T therapy, including pancreaticoduodenectomy and adjuvant chemotherapies, as well as multiple metastases. **(B)** Postoperative pathology revealed pancreatic ductal adenocarcinoma. On the left is hematoxylin and eosin (H&E) staining of tumor tissues (original magnification 100×), in the middle is immunohistochemical (IHC) staining for claudin 18.2 (original magnification 100×) and on the right is IHC staining for claudin 18.2 (original magnification 400×).

The 72-year-old man underwent pancreaticoduodenectomy due to a bile duct space-occupying lesion and the elevated levels of carbohydrate antigen 19-9 (CA19-9) in Queen Mary Hospital, Hong Kong on June 11, 2021. The postoperative pathology indicated pancreatic ductal adenocarcinoma (pT2N2M0, stage III) (**Figure 1B**). Then, he received 8 cycles of gemcitabine plus capecitabine regimen as postoperative chemotherapy until February 2022. The serum levels of CA19-9 continued to rise and reached over 100 u/mL during adjuvant chemotherapy, but no signs of tumor recurrence were observed on CT scans. However, magnetic resonance imaging (MRI) showed liver metastasis and peritoneal metastasis on August 5, 2022, which indicated disease progression. Therefore, the chemotherapy regimen was adjusted to a combination of tegafur, gimeracil, and oteracil potassium. Unfortunately, the left paratracheal lymph nodes of the patient were enlarged on September 20, 2022, which suggested that PC cells metastasized to cervical lymph nodes. Then, the patient was referred to our department for further treatment of metastatic PC, and he was treated with 2 cycles of FOLFIRINOX regimen (fluorouracil, leucovorin, irinotecan, and oxaliplatin) since October 1, 2022 (**Table 1**). During FOLFIRINOX chemotherapy, the patient experienced fatigue, nausea, vomiting, weight loss, neutropenia, and anemia. Due to poor tolerability, the patient refused to continue FOLFIRINOX regimen. Given tumor recurrence and metastasis after multi-line therapies (**Figure 1A**), the patient and his family strongly requested participation in clinical trials of CAR-T cell therapy. Therefore, immunochemistry was performed to detect claudin18.2 expression in tumor tissues, which showed high levels of claudin18.2 expression (positive tumor cell rate ≥70%) (**Figure 1B**). Therefore, the patient was enrolled in a clinical trial of claudin18.2-targeted CAR-T cell therapy (NCT05620732).

**Table 1:** The general clinical characteristics of the patient.

The characteristics of the patient	
Age	72
Sex	male
Diagnosis	pancreatic ductal adenocarcinoma (pT2N2M0)
Stage	stage III
Time from initial diagnosis	2 years
Prior therapies	pancreaticoduodenectomy, gemcitabine plus capecitabine regimen, a combination of tegafur, gimeracil, and oteracil potassium (S-1), FOLFIRINOX regimen (fluorouracil, leucovorin, irinotecan, and oxaliplatin)

**Figure 2:**
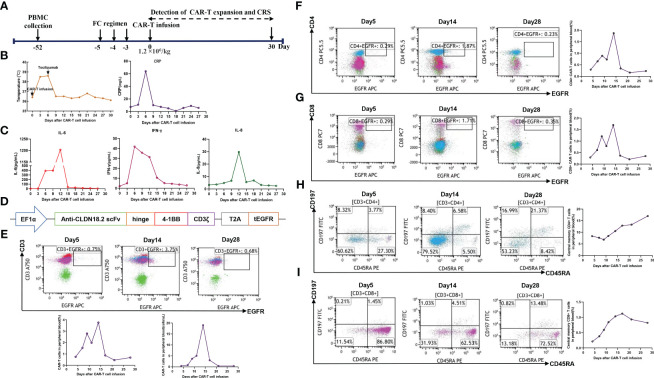
**(A)** Timeline of patient preparation and CAR-T cell therapy from leukapheresis to 1 month after claudin18.2-targeted CAR-T cell infusion. **(B)** Changes in body temperature and the levels of CRP at different time points after CAR-T cell infusion. **(C)** The levels of IL-6, IL-8, and IFN-γ in serum at different time points after CAR-T cell infusion. **(D)** The structure of claudin18.2-targeted CAR. **(E)** The percentage of claudin18.2-targeted CAR-T cells in peripheral blood at different time points. The plot was gated on peripheral blood lymphocytes. **(F)** The percentage of CD4+ CAR-T cells in peripheral blood at different time points. The plot was gated on peripheral blood lymphocytes. **(G)** The percentage of CD8+ CAR-T cells in peripheral blood at different time points. The plot was gated on peripheral blood lymphocytes. **(H)** The percentage of central memory CD4+ T cells in peripheral blood. The plot was gated on peripheral blood CD4+ lymphocytes. **(I)** The percentage of central memory CD8+ T cells in peripheral blood. The plot was gated on peripheral blood CD8+ lymphocytes.

**Figure 3:**
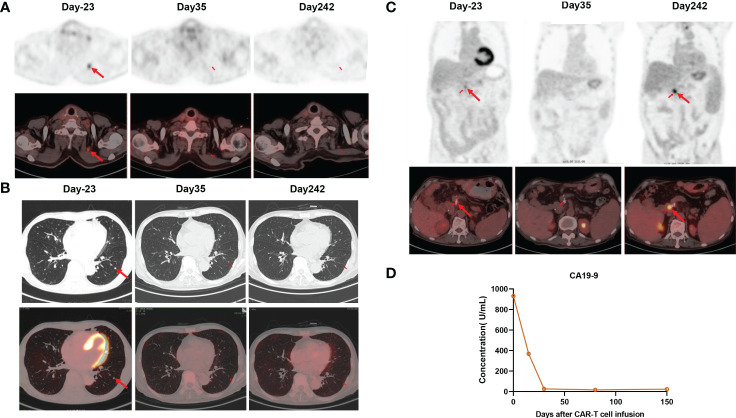
**(A)** PET/CT showed the disappearance of cervical lymph node metastasis 1 month after claudin18.2-targeted CAR-T cell therapy, and it remained in remission 8 months after CAR-T cell infusion. **(B)** PET/CT showed the disappearance of lung metastasis 1 month after claudin18.2-targeted CAR-T cell therapy, and it remained in remission 8 months after CAR-T cell infusion. **(C)** PET/CT showed tumor recurrence at the surgical resection site 8 months after CAR-T cell infusion **(D)** The levels of CA19-9 were markedly decreased after CAR-T cell infusion.

**Figure 4:**
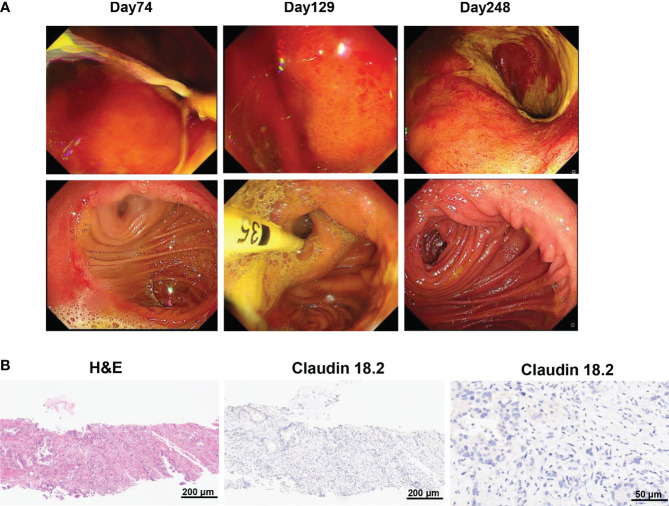
**(A)** Gastroscopy revealed gastric mucosal injury 2 and 4 months after claudin18.2-targeted CAR-T cell therapy, including diffuse gastric mucosal edema and gastric mucosal denudation. The mucosal injury of gastric and duodenal anastomoses as well as gastric mucosal edema was significantly alleviated 8 months after CAR-T cell infusion. **(B)** Biopsy of tumor tissues showed claudin18.2-negative relapse 8 months after claudin18.2-targeted CAR-T cell therapy. On the left is H&E staining of tumor tissues 8 months after CAR-T cell therapy (original magnification 100×), in the middle is IHC staining for claudin 18.2 8 months after CAR-T cell therapy (original magnification 100×) and on the right is IHC staining for claudin 18.2 8 months after CAR-T cell therapy (original magnification 400×).

To manufacture claudin18.2-targeted CAR-T cells, autologous T cells were collected from the patient’s peripheral blood on September 30, 2022, and then genetically engineered to express a chimeric antigen receptor (CAR) which contains a humanized anti-claudin18.2 single-chain variable fragment (scFv) (**Figure 2D**). Then, the patient received lymphodepleting chemotherapy with FC regimen (fludarabine 50 mg/m^2^ day -5 to day -3, cyclophosphamide 400 mg/m^2^ day -5 to day -3) on November 16, 2022. During lymphodepletion preconditioning, fever, chills, and upper abdominal pain were observed, but no abnormalities were detected in blood culture and hepatic function, as well as no signs of acute pancreatitis. After empiric anti-infective therapy and supportive care, the patient’s clinical condition was improved. Given the advanced age and poor tolerability of the patient, the patient was treated with a single infusion of claudin 18.2-targeted CAR-T cells at a dose of 1. 2 × 10^6^ cells/kg on November 21, 2022 (**Figure 2A**). The patient experienced fever 3 days after CAR-T cell infusion (**Figure 2B**), accompanied by fatigue, episodic upper abdominal pain, and nausea. The levels of serum IL-6 was significantly elevated and reached 389.6 pg/mL on day 6 (**Figure 2C**), which suggested grade 2 CRS. Therefore, tocilizumab was utilized to alleviate inflammatory responses. The patient developed diarrhea on day 9, which was controlled by symptomatic treatment, such as gentamicin and antidiarrheal drugs, as well as maintaining water and electrolyte balance. CAR-T cell expansion reached peak levels of 3.75% with 19/μL in peripheral blood on day 14 (**Figure 2E**), and the expansion of CD4 CAR-T cells and CD8 CAR-T cells both reached peak blood levels on day 14 (**Figures 2F, G**). Surprisingly, the levels of the tumor marker CA19-9 were significantly decreased from 1100 U/mL to 25.85U/mL and reached normal range 1 month after CAR-T cell infusion (**Figure 3D**), and PET-CT showed the complete disappearance of tumor lesions, including cervical lymph node metastasis and lung metastasis (**Figures 3A, B**). After clinical assessment, the patient achieved a complete response 1 month after claudin 18.2-targeted CAR-T cell therapy. In addition, the proportion of central memory CD4+ T cells was markedly increased 1 month after claudin 18.2-targeted CAR-T cell therapy (**Figure 2H**), but the proportion of central memory CD8+ T cells reached peak levels on day 17 and then experienced a decline (**Figure 2I**). CA19-9 maintained normal levels 5 months after CAR-T cell therapy (**Figure 3D**), and PET-CT scan revealed no recurrence of neck and lung lesions 8 months after CAR-T cell therapy (**Figures 3A, B**). Unfortunately, PET-CT showed tumor recurrence in situ 8 months after CAR-T cell infusion (**Figure 3C**), and biopsy of tumor tissues showed claudin 18.2-negative (**Figure 4B**). Of note, the gastroscopy and biopsy revealed diffuse gastric mucosal edema, hyperemia, and denudation, accompanied by a large number of lymphocyte infiltration 2 and 4 months after claudin18.2-targeted CAR-T cell therapy (**Figure 4A**), which were partially alleviated by proton pump inhibitors, prokinetic drugs, and gastric mucosal protectants. In addition, a nasogastric tube was used for nutritional support, and the patient returned to free feeding now (**Figure 4A**).

## 3 Methods

### 3.1 Histopathological examination and Immunohistochemistry

For histopathological examination, tumor tissues were fixed in 4% paraformaldehyde for 24 hours. Then, paraffin-embedded tissues were cut into 4 μm thick slices and stained with hematoxylin and eosin (H&E). To detect the level of claudin18.2 expression, these paraffin sections were deparaffinized in xylene and then rehydrated in graded alcohols. After antigen repair with the citrate acid repair solution, endogenous peroxidase enzyme was inactivated with 3% H2O2 solution. After blocking with 5% BSA, slides were incubated with anti-claudin 18.2 primary antibody (Rabbit, 1:500, abcam) and a biotinylated secondary antibody (goat, 1:500, Yeasen). Finally, slides were placed in a DAB chromogenic solution for 10 minutes and counterstained with hematoxylin. These sections were visualized under an optical microscope (Carl Zeiss, Germany), and the total number of PC cells and the number of claudin18.2-positive PC cells were counted. Afterwards, the percentage of claudin18.2-positive cells was calculated, the claudin18.2-positive cell rate = the number of claudin18.2-positive PC cells/the total number of PC cells (×100%).

### 3.2 CAR-T cell manufacture

Peripheral blood mononuclear cells (PBMCs) were collected by apheresis and further isolated and purified by Ficoll density gradient centrifugation. Then T cells were isolated and activated by CD3/28 Dynabeads. After activation for 2 days, the lentiviral vector encoding claudin18.2 CAR was applied for the T cells transduction. The CAR structure consists of a humanized anti-claudin18.2 single-chain variable fragment (scfv), a CD8α hinge region, a 4-1BB co-stimulatory domain and a CD3ζ signaling domain. A truncated, nonfunctional epidermal growth factor receptor (tEGFR) was added at the end of CAR structure with a T2A linker. The tEGFR was used as a marker for transduction and safety switch for anti-EGFR antibody-mediated cell ablation. The CAR-T cells were further expanded in X-vivo medium containing 10ng/ml IL-7, 5ng/ml IL-15, 30ng/ml IL-21 till the cell quantity reached the dose requirement.

### 3.3 Flow cytometry

To detect claudin 18.2-targeted CAR-T cell expansion, the peripheral blood samples (8 mL) were collected in EDTA tubes at different time points. After red blood cell lysis, the remaining cells from each tube were stained with anti-human antibodies, including EGFR-APC (Biolegend, 52906), CD3- APC/Cyanine7 (Biolegend, 300426), CD4-PerCP/Cyanine5.5 (Biolegend, 300530), CD45RA-FITC (Biolegend, 304106), CD197-PE (Biolegend, 353204), and CD8- PE/Cyanine7 (Biolegend, 344712) for flow cytometry analysis. Because claudin 18.2-targeted CAR was integrated with truncated human epidermal growth factor receptor (tEGFR), these claudin 18.2-targeted CAR-T cells could be directly detected by CD3-APC/Cyanine7 and tEGFR-APC antibodies.

## 4 Discussion

PC is a highly malignant digestive system tumor, and thought to be the second most common cause of cancer-related mortality by 2030 (1). Currently, conventional therapeutic strategies confer limited clinical benefits to PC patients. In addition, PC has a high propensity for local invasion and distant metastasis. Therefore, the treatment of PC remains to be a great clinical challenge. At present, novel tumor-specific immunotherapy is being explored to improve the prognosis of advanced or metastatic PC, such as CAR-T cell therapy and T cell receptor-engineered T (TCR-T) cell therapy (9, 11, 12). CAR-T cell therapy is a new type of adoptive T cell immunotherapy, and it has achieved impressive success in R/R B cell malignancies. However, CAR-T-cell therapy showed limited efficacy in solid tumors. There are several factors which contributes to the unsatisfactory outcomes of CAR-T cell therapy in solid tumors. On the one hand, due to tumor antigen heterogeneity in solid tumors, there is a lack of special targets for CAR-T therapy and antigen-negative relapse after CAR-T therapy is more likely to occur. On the other hand, the tumor microenvironment (TME) in solid tumors is complex and exhibits the immunosuppressive properties, which could impair the anti-tumor activities of CAR-T cells and inhibit the infiltration of these cytotoxic T cells (13, 14).

Despite the current limitations of CAR-T cell therapy in solid tumors, several targets for CAR-T cell therapy in advanced or metastatic PC have been explored in the clinic to date, such as claudin18.2, mesothelin (MSLN), epidermal growth factor receptor (EGFR) (15, 16). Besides, there are also many other targets which are being investigated in preclinical studies, including carcinoembryonic antigen (CEA), tumor-associated mucin 1 (tMUC1), growth arrest-specific protein 6 (GAS6), protease-activated receptor 1 (PAR1), glucose-regulated protein 78 KD (GRP78), and trophoblastic cell surface antigen 2 (Trop2) (17–21). Encouragingly, a recent clinical trial demonstrated that claudin18.2-targeted CAR-T cell therapy has promising efficacy in gastrointestinal cancers with an acceptable safety profile, particularly in gastric cancer (11). In the present study, due to tumor recurrence and metastasis after multi-line chemotherapies and the high expression of claudin18.2 in tumor tissues, the patient was enrolled in the clinical trial of claudin18.2-targeted CAR-T cell therapy after a comprehensive assessment. Given few studies on claudin18.2-targeted CAR-T cell therapy in PC and the advanced age of the patient, the patient received the infusion of claudin18.2-targeted CAR-T cells at a dose of 1.2×10^6^/kg on November 21, 2022, which was a relatively lower dose compared with the total dose of 2.5 × 10^8^ cells reported in other studies (9, 11). However, the patient also achieved an impressive response 1 month after the infusion of claudin18.2-targeted CAR-T cells. The levels of the tumor marker CA19-9 were remarkably decreased 1 month after claudin18.2-targeted CAR-T cell therapy and maintained within normal range at 5 months follow-up (**Figure 3D**). Furthermore, all tumor lesions disappeared 1 month after CAR-T cell infusion, and neck and lung lesions maintained remission for up to 8 months (**Figures 3A, B**). These indicates that claudin18.2-targeted CAR-T cells could effectively home to tumor sites and eliminate claudin18.2-postive PC cells. Unfortunately, the patient developed severe gastric mucositis and presented with gastrointestinal symptoms during CAR-T cell therapy, such as poor appetite, nausea, vomiting, and abdominal pain. Due to the expression of claudin 18.2 on normal gastric mucosal cells, while claudin18.2-targeted CAR-T cells kill tumor cells, it may attack normal gastric mucosal cells as well, which was consistent with gastrointestinal adverse effects reported in previous study (11).

The median survival time of patients with advanced PC is 4-8 months (22, 23). Although the patient eventually experienced antigen-negative relapse 8 months after claudin18.2-targeted CAR-T cell therapy, the present study showed the excellent efficacy of claudin18.2-targeted CAR-T cell therapy in metastatic PC. Till now, the patient has survived for 12 months following claudin18.2-targeted CAR-T cell therapy. In a preliminary clinical trial, all 5 patients with PC who were treated with claudin18.2-targeted CAR-T cells experienced disease progression after at least 3 months of follow-up (11). In addition, EGFR and MSLN are also highly expressed in PC cells, and these targets have been explored in clinical trials (15, 16). In a phase 1 clinical trial, 16 patients were treated with anti-EGFR CAR-T cells, and 4 patients achieved partial response for 2-4 months and 8 patients achieved disease stabilization for 2-4 months (15). In another phase 1 clinical trial, 6 patients with refractory PC received MSLN-specific CAR-T cell therapy, and only 2 patients achieved disease stabilization with progression-free survival times of 3.8 and 5.4 months (16). Adverse events were manageable and reversible in these two clinical trials (15, 16). Compared with these two phase 1 clinical trials, the patient in our study achieved deeper and longer duration of remission. In the present study, there are several factors which may facilitate the efficacy of claudin18.2-targeted CAR-T cell therapy. For example, the primary tumor was removed by surgery and metastatic lesions were less than 3cm, which avoided the formation of the immunosuppressive tumor microenvironment. Of note, it is also necessary to prevent gastric mucosal injury due to the on-target off-tumor toxicity mediated by claudin18.2-targeted CAR-T cell therapy, such as optimizing the structure of claudin18.2-targeted CAR-T cells, utilizing drugs with protective roles in gastric mucosa, and adjusting the dose of infused CAR-T cells (11).

## 5 Conclusion

In conclusion, the present study reported a case of complete remission of advanced PC after claudin18.2-targeted CAR-T cell therapy, accompanied by manageable adverse effects, which indicates that claudin18.2-targeted CAR-T cell therapy might be a highly effective therapeutic strategy for advanced PC. Considering antigen-negative relapse 8 months after claudin18.2-targeted CAR-T cell therapy, there is a great need to identify novel tumor-specific surface antigens for the treatment of advanced or metastatic PC. In addition, studies on the efficacy and safety of claudin18.2-targeted CAR-T cell therapy in PC are still in the preliminary stages at present, which needs to be further confirmed in future large-scale clinical trials.

## Data availability statement

The original contributions presented in the study are included in the article/**Supplementary Material**. Further inquiries can be directed to the corresponding authors.

## Ethics statement

The studies involving humans were approved by Shenzhen University General Hospital. The studies were conducted in accordance with the local legislation and institutional requirements. The participants provided their written informed consent to participate in this study. Written informed consent was obtained from the individual(s) for the publication of any potentially identifiable images or data included in this article.

## Author contributions

GZ: Conceptualization, Writing – original draft, Writing – review & editing. XZ: Conceptualization, Formal analysis, Validation, Writing – original draft, Writing – review & editing. ZG: Data curation, Formal analysis, Resources, Writing – original draft. YG: Formal analysis, Methodology, Project administration, Writing – review & editing. BZ: Formal analysis, Methodology, Project administration, Writing – review & editing. XL: Methodology, Resources, Visualization, Writing – review & editing. LC: Investigation, Methodology, Project administration, Writing – review & editing. JQ: Formal analysis, Methodology, Resources, Writing – review & editing. CY: Investigation, Methodology, Software, Writing – review & editing. LW: Investigation, Resources, Supervision, Writing – review & editing. YL: Resources, Writing – review & editing, Writing – original draft. LY: Funding acquisition, Resources, Supervision, Writing – review & editing.
