# Supplementary figures and images for: Complete remission of advanced pancreatic cancer induced by claudin18.2-targeted CAR-T cell therapy: a case report
Source: Front Immunol. 2024 Feb 29;15:1325860. doi: 10.3389/fimmu.2024.1325860 (PMC10937427; doi:10.3389/fimmu.2024.1325860)

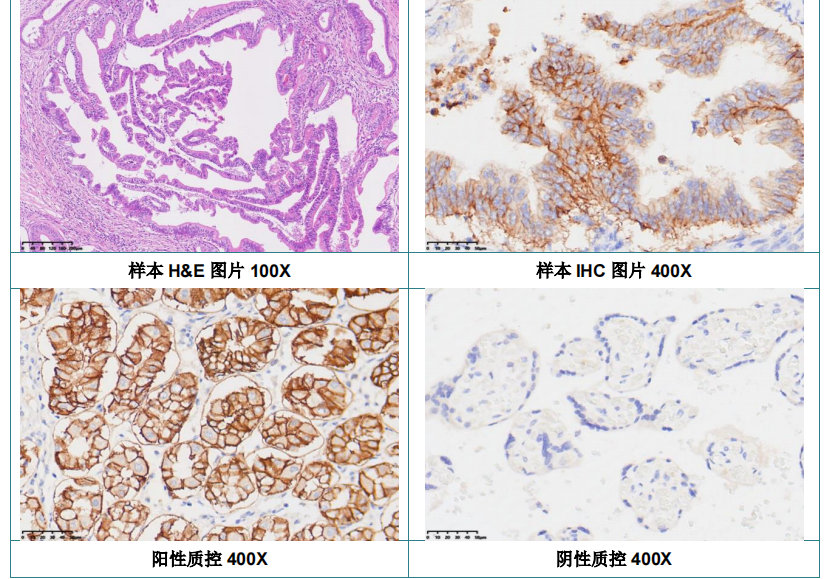

Supplement: Supplementary file 1 [file Image_1.tif]
